# Birth injury in breech delivery: a nationwide population-based cohort study in Finland

**DOI:** 10.1007/s00404-022-06772-1

**Published:** 2022-09-08

**Authors:** Maiju Kekki, Topias Koukkula, Anne Salonen, Mika Gissler, Hannele Laivuori, Tuomas T. Huttunen, Kati Tihtonen

**Affiliations:** 1 Department of Obstetrics and Gynecology, Tampere University Hospital, Elämänaukio 2, 33520 Tampere, Finland; 2 Center for Child, Adolescent and Maternal Health Research, Faculty of Medicine and Health Technology, Tampere University, Tampere, Finland; 3 Faculty of Medicine and Health Technology, Tampere University, Tampere, Finland; 4 Department of Orthopaedics and Traumatology, Tampere University Hospital, Tampere, Finland; 5 Department of Pediatric and Adolescent Surgery, Tampere University Hospital, Tampere, Finland; 6 Department of Knowledge Brokers, Finnish Institute for Health and Welfare, Helsinki, Finland; 7 Department of Molecular Medicine and Surgery, Karolinska Institute and Region Stockholm, Academic Primary Health Care Centre, Stockholm, Sweden; 8 Institute for Molecular Medicine Finland, Helsinki Institute of Life Science, University of Helsinki, Helsinki, Finland; 9 Tampere University Heart Hospital, Tampere University Hospital, Tampere, Finland

**Keywords:** Vaginal breech delivery, Birth injury, Brachial plexus palsy, Birthweight, Epidemiology, Population-based study

## Abstract

**Purpose:**

Previous studies have examined the optimal mode of breech delivery extensively, but there is a scarcity of publications focusing on the birth injuries of neonates born in breech presentation. This study aimed to examine birth injury in breech deliveries.

**Methods:**

In this retrospective register-based nationwide cohort study, data on birth injuries in vaginal breech deliveries with singleton live births were compared to cesarean section with breech presentation and cephalic vaginal delivery between 2004 and 2017 in Finland. The data were retrieved from the National Medical Birth Register. Primary outcome variables were severe and mild birth injury. Incidences of birth injuries in different gestational ages and birthweights were calculated in different modes of delivery. Crude odds ratios of risk factors for severe birth injury were analyzed.

**Results:**

In vaginal breech delivery (*n* = 4344), there were 0.8% of neonates with severe birth injury and 1.5% of neonates with mild birth injury compared to 0.06% and 0.2% in breech cesarean section (*n* = 16,979) and 0.3% and 1.9% in cephalic vaginal delivery (*n* = 629,182). Brachial plexus palsy was the most common type of injury in vaginal breech delivery. Increasing gestational age and birthweight had a stronger effect on the risk for injury among cephalic vaginal deliveries than among vaginal breech deliveries.

**Conclusion:**

Birth injuries were rare in vaginal breech deliveries. The incidence of severe birth injury was two times higher in vaginal breech delivery compared to cephalic vaginal delivery. Brachial plexus palsy was the most common type of injury in vaginal breech delivery.

**Supplementary Information:**

The online version contains supplementary material available at 10.1007/s00404-022-06772-1.

## What does this study add to the clinical work

**Table:** 

In vaginal breech delivery, birth injuries are rare, but brachial plexus palsy is more common than in cephalic vaginal delivery.	

## Introduction

Approximately two to three percent of neonates diagnosed with birth injury [1, 2] are also at increased risk for other morbidities, such as hypoxic-ischemic encephalopathy, seizures, and death [1]. Concern has been raised about the risks for neonates in breech presentation, especially in vaginal delivery (VD), and the risks for the mother and subsequent pregnancies associated with cesarean section (CS) in term [3] and preterm delivery [4–6].

The risk for birth injury in breech presentation is considered comparable to that of cephalic vaginal delivery (cephalic VD) [7, 8], as the reported incidence of birth injury in singleton vaginal breech delivery at term varies from 0.3% to 7.4% [7–11]. In addition, the incidence of birth injury in breech CS has been reported to be between 0.2% and 0.9% [7, 8, 10, 11].

The incidence of morbidity and mortality of neonates after 37^+0^ weeks of gestation with breech presentation was higher after an attempt of VD than after planned CS in two large population-based studies [8, 10] and a randomized multicenter trial (the Term Breech Trial) [11]. Similarly, a systematic review and meta-analysis of non-randomized studies of preterm neonates concluded, and a retrospective cohort study of extremely preterm neonates observed that CS was associated with reduced neonatal mortality [4, 12]. Nevertheless, there is observational evidence showing that VD can be safe with the proper selection of women for both term [13, 14] and preterm neonates with breech presentation [15–17].

A considerable amount of literature has been published on morbidity and mortality rates, whereas only a few studies have focused on birth injuries among neonates born in breech presentation. Since the incidence of birth injuries has been described to be relatively low, a large nationwide register was chosen as a study cohort. This study aims to examine the type and rate of birth injuries in vaginal breech deliveries (breech VD) compared to CS with breech presentation (breech CS) and cephalic vaginal deliveries (cephalic VD) in Finland, where breech VD in selected women is still a common practice. We also aim to describe the incidence of birth injuries in different gestational weeks and explore the risk factors involved, especially those associated with severe birth injury in different types of delivery.

## Materials and methods

This nationwide population-based cohort study was conducted using data from the Finnish Medical Birth Register (MBR) and the Care Register for Health Care. Both registers are maintained by the Finnish Institute for Health and Welfare. All Finnish hospitals are required to report clinical data to these national registries. The MBR includes data on pregnancies, deliveries, and information on the health of neonates. The data are completed by information obtained from the Central Population Register and the Cause-of-Death Register. The Care Register for Health Care contains information on patient diagnoses and operations performed during the hospital stay. The coverage and accuracy of these registers have been shown to be excellent [18, 19].

**Fig. 1:**
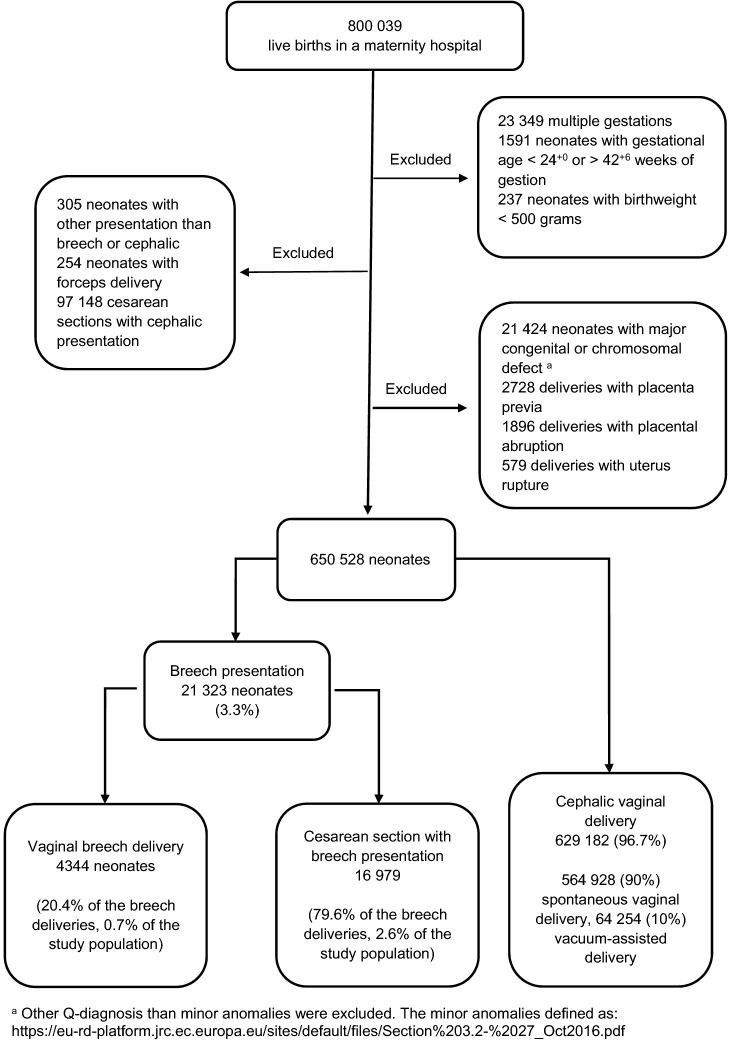
Flowchart of the study population

The study period was from 2004 to 2017, and it focused on singleton breech deliveries that resulted in a live birth. Breech VD and breech CS were studied separately. Planned and unplanned CS were analyzed together (breech CS) since birth injuries were infrequent after CS. Neonates with cephalic presentation born by spontaneous vaginal delivery or vacuum-assisted delivery formed a cephalic VD group, which was used for comparison. Forceps deliveries were excluded as they were rare (254/650,528 neonates), and the presentation of the neonate could not be reliably defined in all cases. Figure 1 presents a flowchart of the study population.

In Finland, five universities with medical faculties offer the education of medical doctors and trainees in gynecology and obstetrics. The management of breech delivery is included in the curriculum of gynecology and obstetrics. There are no national guidelines for term breech pregnancies. However, according to an inquiry addressed to the tertiary level obstetrics centers in Finland, there are common well-established clinical practices for managing breech pregnancies and deliveries after 37^+0^ weeks of gestation: Breech VD is an option if the mother is motivated to vaginal delivery, the estimated fetal weight is < 4000 g, and the fetus is in a frank, complete, or incomplete breech position with the head in a flexed position during the delivery. Often, adequate measurements of the maternal pelvis are confirmed by magnetic resonance pelvimetry. CS is preferred if intrauterine growth restriction is suspected, or the fetus is otherwise at high risk for distress during delivery. According to national guidelines for preterm deliveries, CS may lower the risk for morbidity in primiparas before 32 gestational weeks [20]. However, the mode of preterm delivery is individually selected based on obstetric indications. All breech deliveries are guided by experienced gynecologists, and CS is performed if distress of the fetus is suspected or when difficulties occur during delivery.

The two primary outcome variables were severe and mild birth injury. Birth injuries detected during the early neonatal period (0 to 6 days) were coded with the Finnish implementation of the 10th Revision of International Statistical Classification of Diseases and Related Health Problems (ICD-10) codes and retrieved from the MBR. In addition, hospital visits linked to any birth injury diagnosis recorded in the Care Register for Health Care during the first year after birth were included to increase the coverage. Severe birth injury was defined according to Muraca et al. [21] and included intracranial hemorrhage and laceration, severe injury to the central nervous system, subaponeurotic hemorrhage, skull fracture, long bone injury other than clavicle fracture, brachial plexus palsy (BPP), and injury to the liver or spleen. Mild birth injury included all birth injuries other than severe birth injuries. Outcomes were defined as one or more of the injuries described above. Neonates with both severe and mild birth injuries were included in the severe birth injury group. Outcomes for mild and severe birth injuries with ICD-10 codes are listed in Supplementary information, Table S1.

Tables 2 and 3 present the variables included in the final analysis. Diagnosis of type 1 and type 2 diabetes was based on ICD-10 codes retrieved from the MBR (O24.0, E10*, and O24.1, E11*), and gestational diabetes was defined as pathologic 2 h 75 g oral glucose tolerance test (also O24.4, O24.9). Data concerning prepregnancy body mass index (kg/m^2^) were included after 2006, as values from several hospitals were missing for the years 2004 and 2005. Birthweight above + 2 standard deviations (SD) or below − 2 SDs were defined as large for gestational age and small for gestational age standardized for parity, sex, and gestational age in a Finnish population [22]. The use of oxytocin was registered if it was used to induce and/or augment labor.

### Statistical analyses

The incidences of severe and mild birth injury were calculated and stratified by gestational age. Variables were described as frequencies and proportions for categorical variables, and as means and standard deviations or medians and interquartile ranges for continuous variables. Welch two sample *t*-test and Mann–Whitney *U*-test were used for comparisons of continuous variables.

The risk factors for severe birth injury were calculated. The results are presented as odds ratios and risk differences with 95% confidence intervals (CI). Poisson regression model was used to assess the incidences of birthweight and gestational age using the number of cases per gestational weeks/birthweight as an offset term. The model was used separately for mild and severe birth injuries and in different modes of delivery. Regression analysis was limited to birthweight < 4000 g, as the clinical practice in Finland mainly recommend breech VD when the estimated fetal weight is < 4000 g. Statistical analysis was performed using R Statistical Software version 4.0.3.

## Results

In total, 650,528 neonates were included. Of these, 4344 neonates (0.7%) had breech VD, 16,979 neonates (2.6%) had breech CS, and 629,182 neonates (96.7%) had cephalic VD, either spontaneous (90%) or vacuum-assisted (10%) (Fig. 1).

The incidences and frequencies of injured neonates with different birth injuries are presented in Table 1. The incidence of severe birth injury was highest in the breech VD group, whereas mild birth injury was more common in the cephalic VD group. BPP and clavicle fracture were the most frequent injuries after breech VD. In the breech VD group, 28% of injured neonates had BPP (0.6% of live births) and 24% had clavicle fracture (0.5% of live births). After cephalic VD, clavicle fracture (47% of injured neonates, 1.0% of live births) and cephalhematoma (35% of injured neonates, 0.8% of live births) were the most frequent injuries, followed by BPP (12% of injured neonates, 0.3% of live births). BPP accounted for 82% of the severe birth injuries in the breech VD group and 86% of the severe birth injuries in the cephalic VD group. None of the neonates with breech presentation had both clavicle fracture and BPP, whereas 323 neonates in the cephalic VD group had both. There were no intracranial hemorrhage or central nervous system injuries in the breech VD group and very few in the cephalic VD group. Both severe and mild birth injuries were infrequent in breech CS.

**Table 1:** Frequencies and incidences of neonates with severe birth injury, mild birth injury, and different types of birth injury in vaginal breech delivery, cesarean section with breech presentation, and cephalic vaginal delivery

	Vaginal breech delivery *n* = 4344	Cesarean section with breech presentation *n* = 16,979	Cephalic vaginal delivery *n* = 629,182
Frequency of injured neonates (Incidence/100 live births)			
Severe birth injury	33 (0.76)	10 (0.059)	1954 (0.31)
Mild birth injury	63 (1.45)	35 (0.21)	11,722 (1.86)
Any birth injury	96 (2.21)	45 (0.27)	13,676 (2.17)
ICD-10 codes			
P10: Intracranial hemorrhage or laceration	0	1 (0.0059)	78 (0.012)
P11: Other injuries to central nervous system	0	3 (0.018)	33 (0.052)
P12: Injury to scalp	6 (0.14)	0	5538 (0.88)
P13: Injury to skeleton	29 (0.67)	3 (0.018)	6460 (1.03)
P14: Injury to peripheral nervous system	29 (0.67)	8 (0.047)	1715 (0.27)
P15: Other birth injuries	34 (0.78)	31 (0.18)	492 (0.078)

The birthweight of neonates with severe birth injury was 3320 g (SD 483) in the breech VD group and 4071 g (SD 518) in the cephalic VD group. Gestational ages were similar in both groups, 40^+0^ (interquartile range 38^+5^–40^+4^) and 40^+2^ (interquartile range 39^+2^–41^+1^), respectively (Table 2). We found no statistically significant risk factors for severe birth injury in breech VD (Table 3). For neonates in the cephalic VD group, the most important risk factors for severe birth injury were pregestational diabetes and large for gestational age. In the breech VD group, 3% of neonates with severe birth injury were large for gestational age compared with 19% in the cephalic VD group. Conversely, 3% of injured neonates in the breech VD group were small for gestational age in contrast to 0.6% in the cephalic VD group. The use of oxytocin was the only risk factor found for severe birth injury in the breech CS group.

**Table 2:** Background characteristics of women and neonates with severe birth injury and without severe birth injury in vaginal breech delivery, cesarean section with breech presentation, and cephalic vaginal delivery

	Vaginal breech delivery	*P*-value	Cesarean section with breech presentation	*P*-value	Cephalic vaginal delivery	*P*-value
Number of live births	4344		16,979		629,182	
Number of neonates with severe birth injury	33		10		1954	
Age, years (mean, SD)						
Severe birth injury	30.4 (5.13)	0.64	31.6 (6.13)	0.52	30.0 (5.46)	< 0.001
Without severe birth injury	29.9 (4.97)		30.3 (5.25)		29.5 (5.32)	
BMI, kg/m^2^ (mean, SD)						
Severe birth injury	25.9 (5.76)	0.02	27.2 (9.48)	0.37	26.3 (5.46)	< 0.001
Without severe birth injury	23.4 (4.23)		24.2 (4.74)		24.2 (4.71)	
Height, cm (mean, SD)						
Severe birth injury	165.1 (0.08)	0.08	166.9 (5.11)	0.34	164.4 (5.77)	< 0.001
Without severe birth injury	167.0 (0.08)		165.3 (6.14)		165.8 (5.97)	
Gestational age, weeks^+ days^ (median, interquartile range)						
Severe birth injury	40^+0^	0.11	38^+4^	0.41	40^+2^	< 0.001
	(38^+5^–40^+4^)		(37^+4^–39^+4^)		(39^+2^–41^+1^)	
Without severe birth injury	39^+3^ (38^+1^–40^+2^)		39^+1^ (38^+4^–39^+4^)		40^+1^ (39^+2^–40^+6^)	
Birthweight, grams (mean, SD)						
Severe birth injury	3319.8 (482.92)	0.05	3335.3 (513.51)	0.90	4070.7 (518.26)	< 0.001
Without severe birth injury	3146.1 (564.97)		3314.6 (563.37)		3544.8 (484.98)	

Pregnancies with severe birth injury compared to pregnancies without severe birth injury

*BMI* Body mass index, years 2006 to 2017

*SD* standard deviation

*P*-value calculated using Mann–Whitney *U*-test

**Table 3:** Risk factors for severe birth injury

	Vaginal breech delivery	Cesarean section with breech presentation	Cephalic vaginal delivery
Number of live births	4344	16,979	629,182
Injured neonates (freq.)	33	10	1954
BMI ≥ 30 kg/m^2 a^ (freq.)	324	1823	65,809
Rate of injured neonates (%)	15.2	30	21.4
OR (95% CI)	2.12 (0.81–5.54)	3.48 (0.90–13.46)	2.18 (1.95–2.44)
*P*-value (OR)	0.12	0.07	< 0.001
RD (95% CI)	0.008 (− 0.001–0.03)	0.001 (− 2.50–0.004)	0.003 (0.003–0.004)
Multipara (freq.)	2086	5663	373,376
Rate of injured neonates (%)	42.4	40	58.1
OR (95% CI)	0.77 (0.39–1.55)	1.33 (0.37–4.71)	0.91 (0.83–0.99)
*P*-value (OR)	0.47	0.66	0.03
RD (95% CI)	− 0.002 (− 0.007–0.004)	0.0002 (− 0.0006–0.001)	− 0.0003 (− 0.0006 to − 2.89)
Previous cesarean section (freq.)	177	2224	43,822
Rate of injured neonates (%)	9.1	10	10.2
OR (95% CI)	2.34 (0.71–7.75)	0.74 (0.09–5.81)	1.49 (1.29–1.73)
*P*-value (OR)	0.16	0.77	< 0.001
RD (95% CI)	0.01 (− 0.002–0.04)	− 0.0002 (− 0.0008–0.002)	0.002 (0.0009–0.002)
Gestational diabetes (freq.)	450	2258	78,671
Rate of injured neonates (%)	12.1	30	22.4
OR (95% CI)	1.18 (0.41–3.36)	2.79 (0.72–10.8)	1.98 (1.78–2.21)
*P*-value (OR)	0.76	0.14	< 0.001
RD (95% CI)	0.001 (− 0.005–0.02)	0.0009 (− 0.0002–0.003)	0.003 (0.002–0.003)
Type 1 or Type 2 diabetes (freq.)	11	166	2191
Rate of injured neonates (%)	0	0	2.7
OR (95% CI)	0	0	8.0 (6.07–10.54)
*P*-value (OR)	NA	NA	< 0.001
RD (95% CI)	− 0.008 (− 0.01–0.25)	− 0.0006 (− 0.001–0.02)	0.02 (0.02–0.03)
Use of oxytocin (freq.)	2844	505	276,546
Rate of injured neonates (%)	66.7	20	57.5
OR (95% CI)	1.01 (0.49–2.09)	8.17 (1.73–38.55)	1.67 (1.53–1.83)
*P*-value (OR)	0.98	0.008	< 0.001
RD (95% CI)	0.0008 (− 0.006–0.005)	0.003 (0.0006–0.01)	0.002 (0.001–0.002)
Induction of labor (freq.)	670	322	12,846
Rate of injured neonates (%)	18.2	0	29.1
OR (95% CI)	1.2 (0.49–2.92)	0	1.65 (1.5–1.82)
*P*-value (OR)	0.69	NA	< 0.001
RD (95% CI)	0.001 (− 0.004–0.01)	− 0.0006 (− 0.001–0.01)	0.002 (0.001–0.002)
SGA (freq.)	199	666	14,640
Rate of injured neonates (%)	3.0	0	0.6
OR (95% CI)	0.64 (0.09–4.7)	0	0.25 (0.14–0.45)
*P*-value (OR)	0.68	NA	< 0.001
RD (95% CI)	− 0.003 (− 0.008–0.02)	− 0.0006 (− 0.001–0.005)	− 0.002 (− 0.003 to− 0.002)
LGA (freq.)	23	454	11,196
Rate of injured neonates (%)	3.0	10	18.6
OR (95% CI)	6.0 (0.79–45.89)	4.04 (0.51–31.97)	12.74 (11.35–14.30)
*P*-value (OR)	0.08	0.19	< 0.001
RD (95% CI)	0.04 (0.0007–0.2)	0.002 (− 0.0002–0.01)	0.03 (0.03–0.03)

Rate of injured neonates with risk factor of all injured neonates (%). Crude odds ratios (OR) and risk differences (RD) with 95% confidence interval (CI) presented. Neonates with severe birth injury compared to neonates without severe birth injury.

*BMI* Body mass index, years 2006 to 2017. Due to missing data, the total frequency of injured neonates used with BMI calculation was 1836 in cephalic vaginal delivery, *SGA* small for gestational age, *LGA* large for gestational age, *OR *odds ratio, *RD* risk difference, *CI* confidence interval, *freq* frequency

**Fig. 2:**
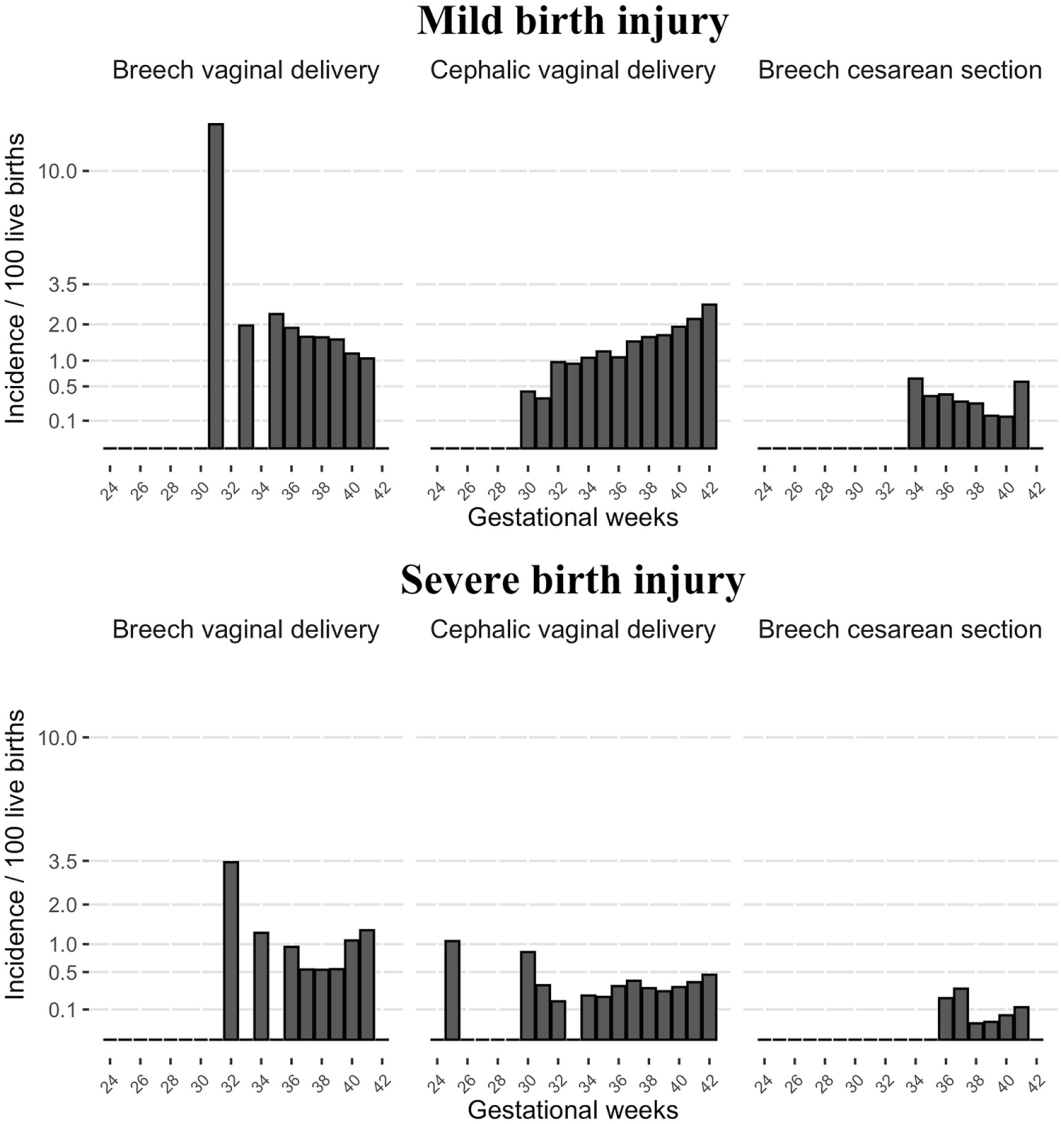
The incidence of mild birth injury (%) and severe birth injury (%) in different gestational weeks in vaginal breech delivery (*n* = 4344), cephalic vaginal delivery (*n* = 629,182), and cesarean section with breech presentation (*n* = 16,979) between 2004 and 2017 in Finland. Incidence presented as square root variant

Between gestational weeks 24^+0^ and 27^+6^ 41% (51/124), 28^+0^ and 31^+6^ 29% (55/187), 32^+0^ and 36^+6^ 30% (500/1654), 37^+0^ and 40^+6^ 19% (3336/18,014), and 41^+0^ and 42^+6^ 30% (402/1344) of fetuses with breech presentation had VD. There were no severe birth injuries, and three neonates (at 31 weeks of gestation) in the breech VD group had a mild birth injury between 24^+0^ and 31^+6^ weeks of gestation. In the cephalic VD group, there was a similar finding of single injuries. After 32 weeks of gestation, the incidence of injury remained stable with some sporadic fluctuation up to 42 weeks of gestation among the breech VD group (Fig. 2). Between gestational weeks 32^+0^ and 36^+6^ 8 mild and 4 severe birth injuries, 37^+0^ and 40^+6^ 48 mild and 24 severe, and 41^+0^ and 42^+6^ 4 mild and 5 severe birth injuries were diagnosed among the breech VD group. Also, in the breech VD group, no association was found in Poisson regression analysis between the incidence of mild birth injury and gestational weeks (estimated increase to incidence of injury for 1 gestational week was 0.96, 95% CI 0.88–1.05) or the incidence of severe birth injury and gestational weeks (1.12, 95% CI 0.93–1.35). In contrast, the incidence of birth injury showed an increasing trend with higher gestational age in the cephalic VD group (mild birth injury: 1.13, 95% CI 1.11–1.14, severe birth injury: 1.07, 95% CI 1.04–1.11), Fig. 2. There was no association between gestational age and incidence of birth injury in breech CS.

**Fig. 3:**
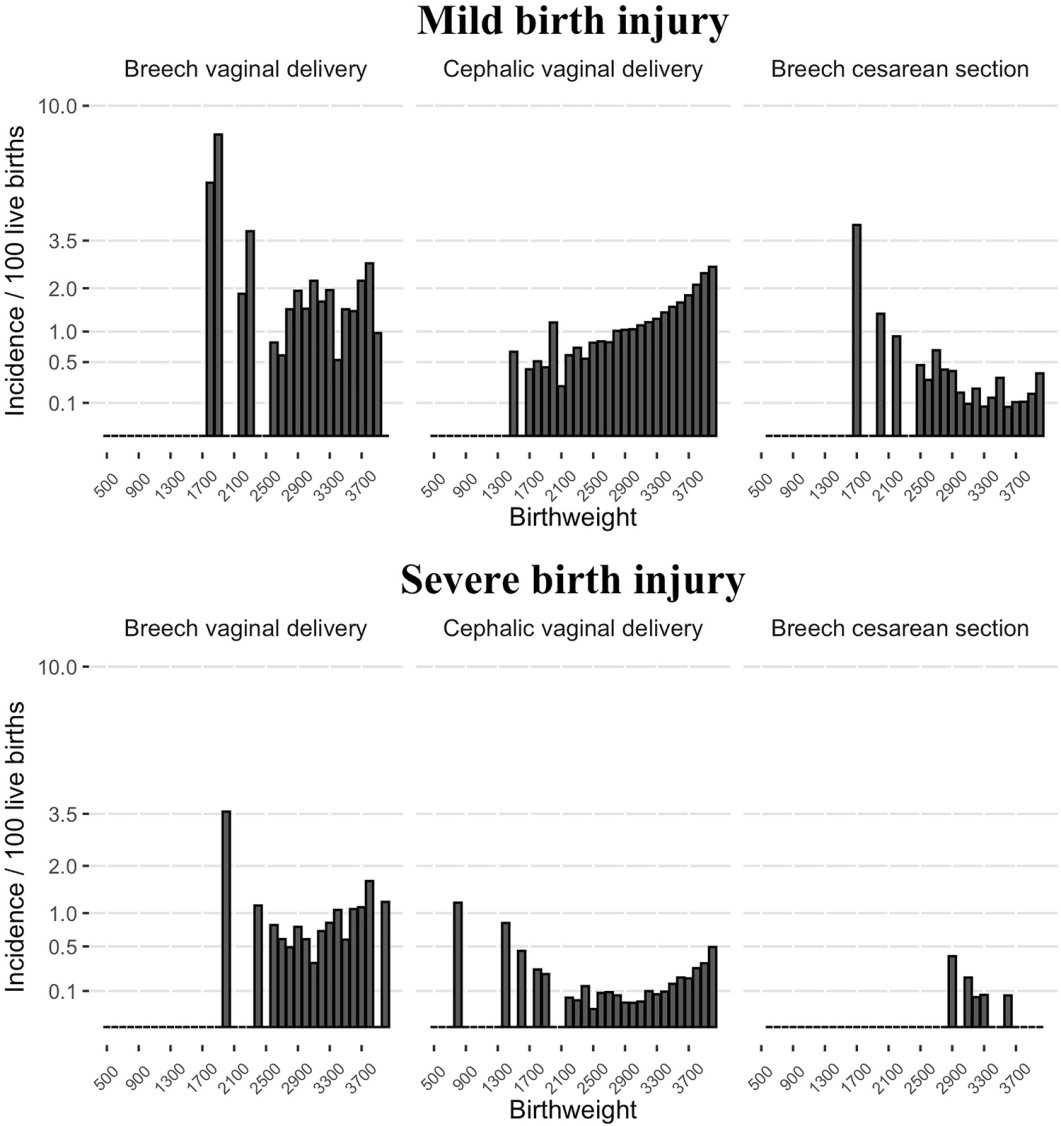
The incidence of mild birth injury (%) and severe birth injury (%) in different birthweight (500–4000 g) in vaginal breech delivery (*n* = 4344), cephalic vaginal delivery (*n* = 629,182), and cesarean section with breech presentation (*n* = 16,979) between 2004 and 2017 in Finland. Incidence presented as square root variant

**Fig. 4:**
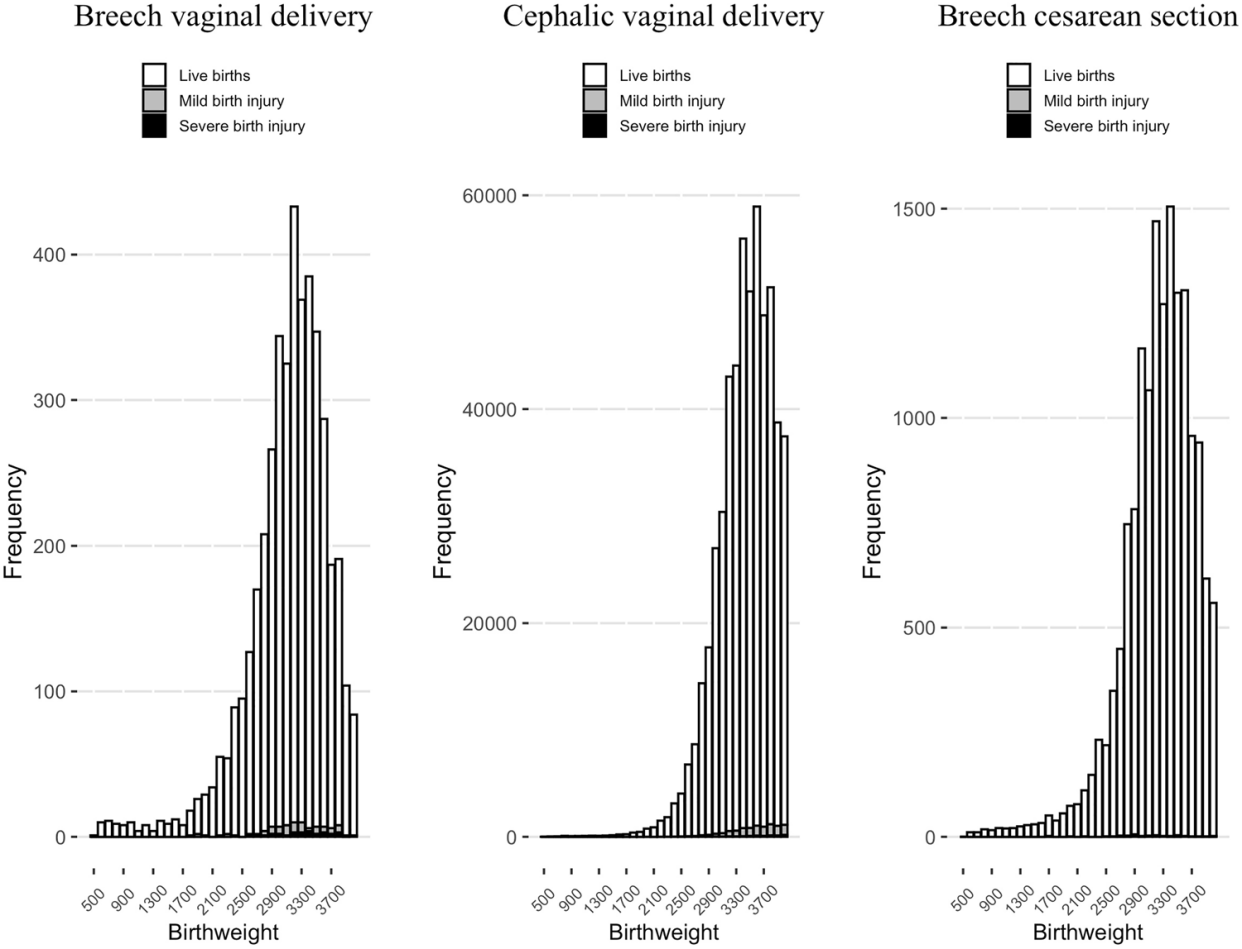
Frequency of live births, mild birth injuries, and severe birth injuries in different birthweight (500–4000 g) in vaginal breech delivery (*n* = 4344), cephalic vaginal delivery (*n* = 629,182), and cesarean section with breech presentation (*n* = 16,979) between 2004 and 2017 in Finland

Furthermore, no association was found in Poisson regression between birthweight (500 g to 4000 g) and incidence of mild birth injury (the estimated increase in incidence of injury for an increase of 100 g in birthweight was 1.01, 95% CI 0.97–1.06) or between birthweight and severe birth injury (1.06, 95% CI 0.98–1.14) in breech VD. In the cephalic VD group, the incidence of mild birth injury (1.09, 95% CI 1.09–1.10) and the incidence of severe birth injury (1.20, 95% CI 1.17–1.22) showed an increasing trend along with higher birthweight. In the breech CS group, however, the incidence of mild birth injury seemed to show a decreasing trend with increasing birthweight (0.94, 95% CI 0.90–1.0) (Fig. 3). There were only three neonates with severe birth injury (incidence 1.63%) and no neonates with mild birth injury and birthweight over 4000 g in the breech VD group (total of 184 neonates) (Fig. 4).

## Discussion

In this population-based study, the incidence of severe birth injury was higher in the vaginal breech delivery group than in the cesarean section with breech presentation group and the cephalic vaginal delivery group. However, the incidence remained low in all groups. A brachial plexus palsy was the most frequent injury in vaginal breech delivery. Perhaps because of the more rigorous selection of women for vaginal delivery, high birthweight did not seem to be as important a risk factor for birth injury in vaginal breech delivery as in cephalic vaginal delivery.

The total incidence of birth injury in breech VD was similar to that previously reported [8, 9, 11]. Surprisingly, there were no intracranial hemorrhage or central nervous system injuries after breech VD. In breech VD, a BPP was the most common injury followed by clavicle fractures. Although breech presentation is a risk factor for BPP [23], few studies have exclusively focused on BPP among neonates in breech presentation [24]. Moreover, it has been suggested that neonates born in breech presentation with BPP have a worse prognosis, a higher rate of bilateral plexus injuries, and a higher rate of concurrent phrenic nerve palsies than neonates born with an injury in cephalic presentation [24]. In our study, no concurrent clavicle fractures were found, and the birthweight of the injured neonates was lower in the breech VD group than in the cephalic VD group. These findings may suggest that BPP in breech VD may be due to unnecessary traction of the shoulders during delivery or difficulties in delivering an entrapped head. However, based on the findings of this study, we are unable to draw a definitive conclusion on this. In addition, we do not know whether the BPP identified in our study population were bilateral or persistent. Spinal cord injuries, of which none were found in our data, have been reported after a difficult head delivery [25]. In Finland, Løvset and Mauriceau maneuvers are most often used to deliver shoulders and head. However, due to the retrospective study design, we do not know which maneuvers if any, were used. International clinical practice guidelines recommend avoiding traction in the active second stage of vaginal delivery, but any specific maneuver is not favored [26]. In future studies, BPP in breech deliveries and difficulties with delivering the head should be specifically assessed.

Risk factors for severe birth injury, mostly representing the risk factors for BPP, found in cephalic VD were comparable to the risk factors reported for BPP in previous studies that mainly concerned neonates in cephalic presentation (fetal macrosomia, maternal diabetes, instrumental vaginal delivery, and shoulder dystocia) [23]. In the present study, we found no risk factors for severe birth injuries in breech VD. This finding may be due to the low number of injuries in the breech VD group. Another possible explanation might be the stricter selection of women for vaginal delivery and the lower threshold for antepartum and intrapartum CS when the fetus is in the breech presentation compared to pregnancies with the fetus in the cephalic presentation. The observed increase in the risk for severe birth injury with the use of oxytocin in the breech CS group is probably attributed to the attempted vaginal delivery.

The incidence of birth injury was low in all gestational ages in neonates with breech presentation, and no evidence was found of an association between gestational age and birth injury. It has been suggested that CS reduces perinatal morbidity and mortality in preterm breech neonates [4, 12, 27], but the improvement in neonatal outcomes is not supported by all researchers [15–17]. The Finnish guideline for preterm deliveries concludes that CS may reduce morbidity and mortality for neonates in breech presentation at < 32 weeks of gestation [20]. Although the low number of injuries in breech deliveries reduces the predictability and accuracy of the regression analysis results, our results suggest that the current clinical policy in Finland to manage preterm vaginal breech deliveries in selected women is acceptable, considering the low risk for birth injuries. Unfortunately, head entrapments are not registered in the MBR, and therefore the number of this rare and feared complication that is associated with preterm breech VD is unknown [15, 16].

Furthermore, the significance of high birthweight remains unclear. The guidelines recommend preferring CS when the estimated birthweight is  > 3800 to 4000 g [28–30], but high birthweight has not been clearly shown to be associated with adverse outcomes [14, 31]. In Finland, there are no national guidelines for managing breech deliveries, although an estimated fetal weight of  < 4000 g is a widely used criterion for attempted vaginal delivery. In the present study, we could not find an association between increasing birthweight or large for gestational age and birth injury in breech VD; however, they were risk factors for severe birth injury in cephalic VD. As previously mentioned, these results regarding breech VD and birthweight may have been affected by the rigorous selection of women and the surveillance of labor in addition to a low number of cases. To summarize, our results suggest that the current Finnish policy of managing breech pregnancies and breech VD up to a birthweight of 4000 g is acceptable, especially concerning birth injuries.

This study provides valuable information on the risks associated with breech deliveries. The strength of this study was the nationwide study population and the long study period that enabled us to study rare incidents such as birth injuries. In Finland, reporting to the registers is mandatory, the medical treatment of pregnancies is homogenous even without national guidelines for breech pregnancies, and the rate of breech VD has remained stable during the twenty-first century [32]. Thus, register data have good national coverage, and the reporting and selection biases are low [18, 19]. Our results were, however, restricted by the retrospective study design in which we are unable to study the intended mode of delivery, and rule out the possibility of variation among coding practices. Some of the most difficult deliveries, with failure to deliver head by traditional maneuvers, may have been excluded due to exclusion of forceps deliveries. Furthermore, even with a large sample size, the number of birth injuries remained modest, and thus limited the statistical power of the results. The simulation-based training of breech deliveries started at the end of the study period in delivery units, and a specific program of simulation training was launched in 2021 [33]. Hopefully, the implementation of the simulation training program improves the training and safety of breech deliveries in the future.

## Conclusion

Our study confirmed that risk for birth injury is low in breech VD and breech CS. Nevertheless, the risk for severe birth injury, specifically BPP, was higher among breech VD than breech CS or cephalic VD. Birth injuries in neonates with breech presentation were sporadic, and no clinically relevant risk factors were found. These findings suggest that careful selection of women is required to ensure safe vaginal breech delivery.

## Supplementary Information

Below is the link to the electronic supplementary material.

Supplementary file1 (PDF 14 KB)
