# Supplementary material for: Birth injury in breech delivery: a nationwide population-based cohort study in Finland
Source: Arch Gynecol Obstet. 2022 Sep 8;308(4):1139–50. doi: 10.1007/s00404-022-06772-1 (PMC10435420; doi:10.1007/s00404-022-06772-1)
Supplement: Supplementary file 1 — Supplementary file1 (PDF 14 KB) [file 404_2022_6772_MOESM1_ESM.pdf]

Supplementary Table 1 Outcome variables

Outcome variables	ICD-10 codes
Severe Birth injury	
Intracranial hemorrhage or laceration	P10.0 - P10.9
Severe central nervous system injury	P11.0 - P11.2, P11.4 - P11.5
Subaponeurotic hemorrhage	P12.2
Skull fracture, long bone injury (excluding clavicle fractures)	P13.0, P13.2, P13.3
Brachial plexus injury	P14.0 - P14.3
Injury to the liver or spleen	P15.0, P15.1
Mild Birth Injury	
Facial nerve and unspecified central nervous system injury	P11.3, P11.9
Cephalhematoma, Chignon, other and unspecified scalp injury	P12.0, P12.1, P12.3 - P12.9
Skull injury, clavicle fracture, other and unspecified injury to skeleton	P13.1, P13.4, P13.8, P13.9
Other and unspecified peripheral nervous system injury	P14.8, P14.9
Sternomastoid, eye, face, external genital, other and unspecified birth injury	P15.2 - P15.9

Birth injury in breech delivery: A nationwide population-based cohort study in Finland.
Archives of Gynecology and Obstetrics.
Kekki M, Koukkula T, Salonen A, Gissler M, Laivuori H, Huttunen TT, Tihtonen K.
Corresponding author: Kekki M. Department of Obstetrics and Gynecology, Tampere University Hospital, Tampere, Finland. Center for Child, Adolescent and Maternal Health Research, Faculty of Medicine and Health Technology, Tampere University, Tampere, Finland. maiju.kekki@pshp.fi
